# Getting Recovery Right After Neck Dissection (GRRAND-F): Mixed-methods feasibility study to design a pragmatic randomised controlled trial

**DOI:** 10.3389/fonc.2023.1110500

**Published:** 2023-03-16

**Authors:** Toby O. Smith, Angela Garrett, Tianshu Liu, Alana Morris, Victoria Gallyer, Bethany A. Fordham, Susan J. Dutton, Mae Chester-Jones, Sarah E. Lamb, Stuart Charles Winter

**Affiliations:** ^1^ Warwick Clinical Trials Unit, University of Warwick, Coventry, United Kingdom; ^2^ School of Health Sciences, University of East Anglia, Norwich, United Kingdom; ^3^ Oxford Clinical Trials Research Unit, Centre for Statistics in Medicine, Nuffield Department of Orthopaedics, Rheumatology and Musculoskeletal Sciences, University of Oxford, Oxford, United Kingdom; ^4^ Nuffield Department of Orthopaedics, Rheumatology and Musculoskeletal Sciences, Medical Sciences Division, University of Oxford, Oxford, United Kingdom; ^5^ University of Exeter Medical School, University of Exeter, Exeter, United Kingdom; ^6^ Nuffield Department of Surgical Sciences, Medical Sciences Division, University of Oxford, Oxford, United Kingdom; ^7^ Oxford Cancer Centre, Churchill Hospital, Oxford University Hospitals NHS Foundation Trust, Oxford, United Kingdom

**Keywords:** head and neck, cancer, shoulder, rehabilitation, surgery

## Abstract

**Objective:**

To determine the feasibility of a randomised controlled trial to estimate the effectiveness and cost-effectiveness of a rehabilitation intervention following neck dissection (ND) after head and neck cancer (HNC).

**Design:**

Two-arm, open, pragmatic, parallel, multicentre, randomised controlled feasibility trial.

**Setting:**

Two UK NHS hospitals.

**Participants:**

People who had HNC in whom a ND was part of their care. We excluded those with a life expectancy of six months or less, pre-existing, long-term neurological disease affecting the shoulder and cognitive impairment.

**Intervention:**

Usual care (standard care supplemented with a booklet on postoperative self-management) was received by all participants. The GRRAND intervention programme consisted of usual care *plus* up to six individual physiotherapy sessions including neck and shoulder range of motion and progressive resistance exercises, advice and education. Between sessions, participants were advised to complete a home exercise programme.

**Randomisation:**

1:1 randomisation. Allocation was based on minimisation, stratified by hospital site and spinal accessory nerve sacrifice. It was not possible to mask treatment received.

**Main outcome measures:**

Primary: Participant recruitment, retention and fidelity to the study protocol and interventions from study participants and staff at six months post-randomisation (and 12 months for those reaching that time-point). Secondary: clinical measures of pain, function, physical performance, health-related quality of life, health utilisation and adverse events.

**Results:**

36 participants were recruited and enrolled. The study achieved five of its six feasibility targets. These included consent - 70% of eligible participants were consented; intervention fidelity - 78% participants discharged completed the intervention sessions; contamination - none - no participants in the control arm received the GRRAND-F intervention and retention - 8% of participants were lost to follow-up. The only feasibility target that was not achieved was the recruitment target where only 36 of the planned 60 participants were recruited over 18 months. This was principally due to the COVID-19 pandemic which caused all research activity to be paused or reduced, with a subsequent reduction in.

**Conclusions:**

Based on the findings a full-trial can now be designed to better understand whether this proposed intervention is effective.

**Clinical Trial Registration:**

https://www.isrctn.com/ISRCTN1197999, identifier ISRCTN11979997.

## Introduction

Annually, head and neck cancer (HNC) is diagnosed in 700,000 people worldwide and over 11,000 in the UK (1, 2). Within the UK, tumours of the oropharynx are the most common and have seen a two-fold increase in incidence over the last 20 years, largely attributed to human papillomavirus (HPV) (3, 4). Over the last 20 years, there has also been a 30% increase in oral cancer; these increases are predicted to continue (1). In addition, there is a significant health burden from thyroid cancer as well as skin cancers which are all predicted to increase in prevalence (1). People affected by HNC are now younger, more active and more ethnically diverse than previous generations of HNC survivors (1).

The treatment pathway for HNC is complex. Surgery and/or radiotherapy and/or chemo-radiotherapy is used to treat the primary tumour. From a surgical perspective, a neck dissection (ND) can be performed. Historically, a ND involved removal of all the lymph nodes as well as potentially key structures such as the spinal accessory nerve, the internal jugular vein and the sternocleidomastoid muscle. While these radical procedures are now relatively uncommon, it is now more common to remove selected lymph node levels that have been defined and preserve key structures (5).

Side-effects from surgery can be substantial, including swallowing problems, neck and shoulder problems, difficulties sleeping, fatigue and anxiety (6, 7). Post-operative complications are common following ND, occurring in 50-100% of patients (8–10). Early complications can include shoulder pain and infection. Late complications may not appear until three months post-treatment and can continue to present over five years (11). These complications include shoulder movement dysfunction, speech, swallowing and musculoskeletal problems such as cervical contracture and muscle wastage (11). Shoulder dysfunction is particularly evident where injury to the accessory nerve occurs during surgery (12). Post-operative psychosocial complications are also common, predominantly being fatigue, anxiety, depression, sleep disturbance and social isolation. Sequelae of shoulder dysfunction and psychosocial complications are strongly associated with reduced return to work. Up to 50% of patients ceasing working due to shoulder disability alone (10, 13).

There is currently no national standard best practice for effective rehabilitation following HNC treatment which involved ND. The 2016 National Institute for Health and Care Excellence (NICE) Clinical Guideline on the management of HNC (8) recommended clinicians “consider progressive resistance training for people with impaired shoulder function, as soon as possible after ND”. The review noted that this evidence was from small trials with a high risk of bias. As such, physiotherapy practice varies across the UK. Rehabilitation in the form of physiotherapy is not routinely available to some patients with HNC, in either in-patient or outpatient settings and when it is offered, it is often not evidence-based (14). Furthermore there remains a gap in knowledge on how to rehabilitate patient’s wider side-effects following surgery for HNC such as fatigue, anxiety, poor sleep and return to work.

There is limited research on whether rehabilitation interventions such as physiotherapy may improve shoulder or neck function, quality of life or reduce complications neck dissection for HNC. Given the health challenge which people following neck cancer for HNC face post-operatively, testing rehabilitation interventions to improve clinical outcomes is therefore valuable. Understanding how feasible it would be to recruit and retain participants and whether a rehabilitation intervention is acceptable and can be delivered are key trail design uncertainties which require to be answered to determine whether a full-trial is appropriate. Given this uncertainty, the aim of this study was to evaluate whether it was feasible to conduct a randomised controlled trial (RCT) to assess the effectiveness of a rehabilitation intervention in improving pain, function and health-related quality of life following ND after HNC.

## Methods

### Study design

A full protocol has been published previously (15). The methods and results of the qualitative sub-study associated with this trial have been previously reported (16).

This study has been reported in accordance with the CONSORT extension for pilot and feasibility studies reporting checklist (17).

This was a two-arm, open, pragmatic, parallel, multicentre, randomised controlled feasibility trial. Participants were recruited from two UK National Health Service (NHS) hospitals by the clinical team once they had been listed for ND surgery for HNC. Recruitment occurred between January 2020 to June 2021. Interventions were delivered in physiotherapy departments within these hospitals.

### Study objectives

We aimed to determine:

1. Recruitment and retention rates from study participants across sites.

2. Potential risks of intervention contamination.

3. Feasibility and acceptability of the intervention from patient and physiotherapist perspectives.

4. Sample size calculation for a definitive trial.

5. Wider experiences and perceptions of the study design from a patient and physiotherapist perspective.

Objective 5 has been previously reported in a qualitative sub-study paper (16).

### Participant eligibility

Participants were eligible if they were adults who had HNC which involved ND as part of their care; were willing to attend the physiotherapy outpatient department (if randomised to the experimental arm), and provided they gave written informed consent. We excluded people whose treatment was palliative (expected survival six months or less), those with a pre-existing, long-term neurological disease affecting the shoulder, for example, hemiplegia and people with cognitive impairment (defined as an Abbreviated Mental Test Score of seven or less) (18). Consented participants were randomised post-surgery.

### Study treatments

#### Usual care group

Usual care was received by both control and experimental intervention groups. This consisted of standard NHS recovery and rehabilitation following NC for HNC including of simple range of motion (ROM) exercises for face, neck and shoulder, respiratory care to promote sputum clearance, breathing control and exercise tolerance, education on body positioning, oral health to reduce food pocketing and pain management advice. All participants on discharge from the in-patient setting received a booklet providing advice on postoperative self-management strategies including exercise, pain management, return to work and activities of daily living. Reflecting usual care, those allocated to the usual care group, once discharged from hospital, were not routinely referred to physiotherapy. This reflects usual practice in the UK NHS service, allowing the design the compare how a different rehabilitation approach (experimental intervention) compares to current service delivery.

#### Experimental group

Participants randomised to this group received the same in-patient rehabilitation programme as participants in the usual care group *PLUS* an individualised rehabilitation programme. As described in full previously (19), this was delivered by a physiotherapist trained in the experimental intervention in an outpatient setting. This was delivered either face-to-face in hospital or virtually. In brief, the intervention permitted physiotherapists to prescribe treatments to address modifiable physical and psychosocial factors associated with poor recovery following HNC surgery. These could include: muscle weakness, limited ROM, reduced sensation, pain and fear avoidance beliefs. Programmes were individualised to contain one, several or all treatment options, dependent on participant’s needs. Participants were provided with a home exercise programme to supplement face-to-face sessions.

The experimental intervention could be delivered over a maximum of six sessions during a six-month period. The first session was aimed to occur within 14 days of surgery. The initial session was up-to 60 minutes in duration with subsequent sessions up to 45 minutes. The physiotherapist, in collaboration with the participant, agreed the spacing of sessions based on need depending on clinical presentation, participant preference and symptoms during adjunctive treatments which may impact on require or capacity to participate in the rehabilitation sessions.

### Data collection

Baseline data were collected prior to randomisation, once consent had been obtained.

Data were clinical and participant-reported and collected using questionnaires at baseline and six months post-randomisation (primary end-point) during routine clinical appointments. Data were also collected for those participants who reach 12-month follow-up during the data collection phase. Data collected is summarised in **Table 1**. The clinical outcome data collected were included for three reasons: (1) to ensure that we were able to assess completion rate and overall study retention for the outcome measures used; (2) to provide the parameters to inform the sample size calculation for a definitive trial, and; (3) to offer a ‘signal’ of treatment efficacy which may infer promise the treatment may be beneficial, offering additional justification for the need for a definitive trial.

**Table 1:** Summary of data collected.

Feasibility outcome data including: • Screening log to determine numbers of eligible patients, including reasons for exclusion/non-participation, recruitment numbers and rate (overall and per site). • Treatment logs to determine treatment protocol adherence, fidelity to control and experimental interventions using, timing and location of intervention delivery (in particular the first session) alongside frequency of physiotherapy contact. • Data CRFs and PROMS to determine follow-up completion rate and overall study retention in each study arm. Clinical data including: • Shoulder pain and function measured using the Shoulder Pain and Disability Index (SPADI) (1, 2). • Pain measured using the SPADI 5-item Pain Sub-scale (2) and a Numerical Rating Scale. • Function measured using the SPADI 8-item Function subscale (2) • Pain medication details and usage relating to head, neck and shoulder. • Chemotherapy and radiotherapy treatment provision. • Health-related quality of life measured using the EQ-5D-5L score (6) and the EORTC questionnaires (C30 (core) (8) and H&N43 (head and neck specific) (9, 10). • Health resource use questionnaire. • Physical performance measures including goniometer-measured shoulder and neck active ROM and hand-held dynamometer-measured grip strength. • Adverse events such as prolonged delayed onset muscle soreness, swelling and wound irritation.

### Randomisation, blinding and allocation concealment

Random allocation was 1:1. Randomisation was performed using a centralised computer randomisation programme provided by Oxford Clinical Trials Research Unit (OCTRU). Research nurses and physiotherapists at recruiting centres assigned participants by accessing the online randomisation programme to adopt a concealed allocation approach. Allocation was based on minimisation, stratified by hospital site and spinal accessory nerve sacrifice.

Due to the nature of the intervention, masking participants or the teams providing interventions was not possible. Investigators taking the clinical measurements were blinded to the intervention.

### Sample size

We originally planned to recruit 60 participants, based on Whitehead et al. (20) and Teare et al’s recommendation (21). This assumed a 10% drop-out. Based on our 2017 data, this was considered realistic from two participating sites where approximately 160 potentially eligible participants were identified in that year. However, recruitment was significantly impacted by the COVID-19 pandemic where both clinical and research activity was halted at intervals during the study period.

### Data analysis methods and progression criteria

To assess the trial feasibility, we calculated the rate of eligible participants who consented to be included in the trial, trial recruitment rate and retention to six months. The flow of participants through the study from identification to screening and then to follow-up was summarised using a CONSORT diagram (17). Availability of data at each follow-up time point were summarised. The baseline comparability of the two intervention groups in terms of minimisation factors and baseline characteristics are described as proportions for categorical variables, and as mean and standard deviation (SD) or median and interquartile range (IQR) for continuous variables depending on the distribution. The number of withdrawals, protocol deviations, losses to follow-up, deaths, adverse events and details of treatment received were summarised by treatment group.

We analysed the clinical outcomes to explore whether there was a ‘signal’ of efficacy for the experimental intervention for the shoulder pain and function measured using the Shoulder Pain and Disability Index (SPADI) (22, 23) and EORTC questionnaires (C30 (core) and H&N43 (head and neck specific) (24, 25) since these assessed the key domains of pain, function and HRQoL. These were analysed on an intention-to-treat (ITT) basis. Treatment differences and 95% confidence intervals (CI) are reported throughout SPADI and EORTC QLQ C30 and H&N43 items were summarised using with mean and SDs presented by treatment arms. A linear model adjusting for baseline factors only was used to estimate the treatment differences between the two arms at the six month follow-up time-point. Due to only a small number of participants reached the 12-month follow-up time-point, the treatment differences calculated at 12 months were only exploratory. No p-values are reported as the study was not powered to test for differences between clinical or patient-reported outcomes.

To determine whether this trial could progress onto a phase III definitive trial, we used pre-specified traffic light stop-amend-go progression criteria (19) and interpretation from the qualitative aspects of the study (16). This was reviewed by the Trial Oversight Committee (TOC) to provide a recommendation on the outcome of feasibility principally based on the results of the progression criteria.

### Study monitoring

A TOC was appointed to independently review data on safety, protocol adherence and trial recruitment.

## Results

### Patient characteristics and treatment

**Figure 1:**
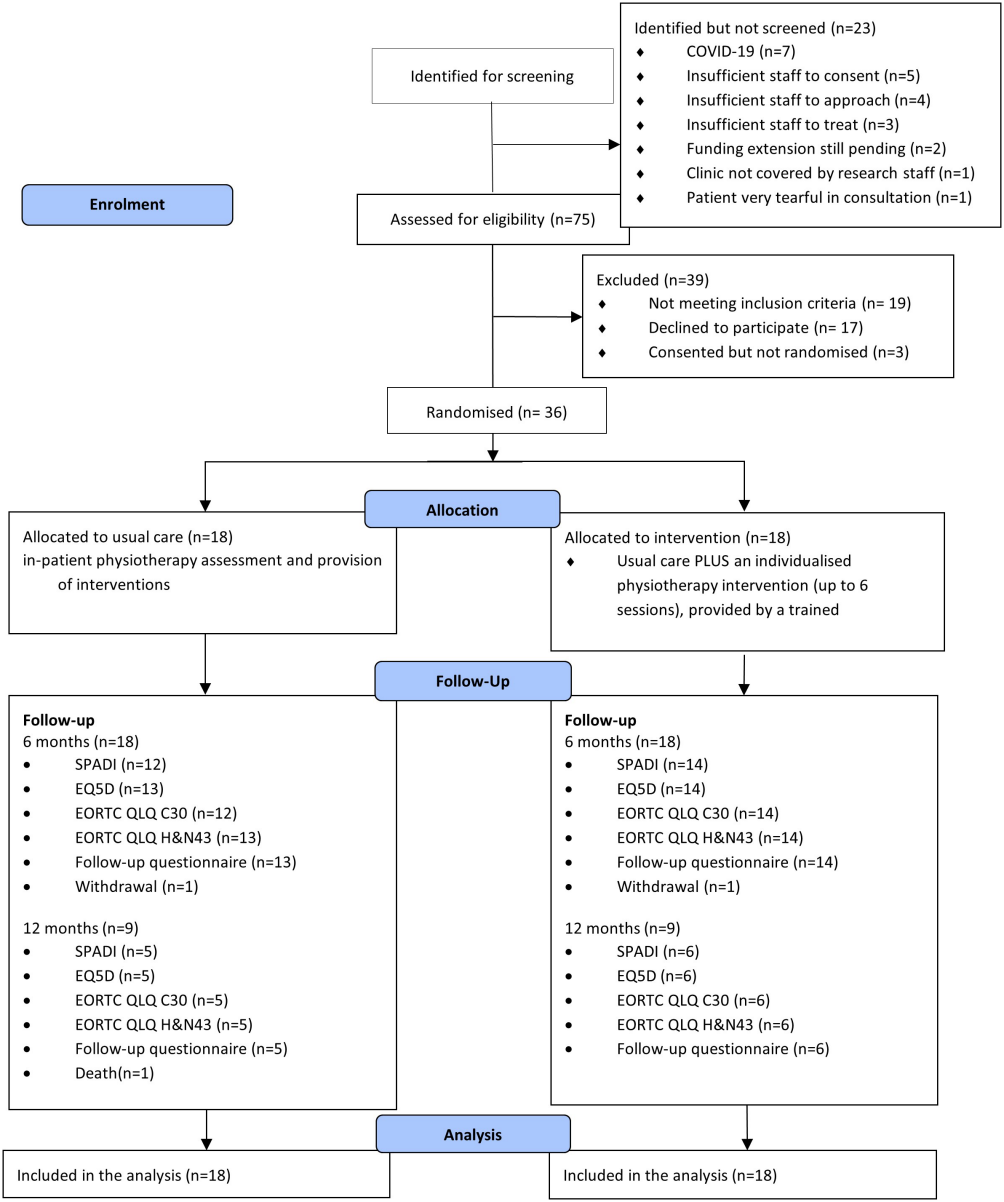
CONSORT diagram from screening to analysis.

Thirty-six participants were recruited. This is summarised in **Figure 1**. The complete list of baseline characteristics in both experimental intervention and control groups is presented in **Table 2**.

**Table 2:** Baseline participant characteristics.

	Intervention (N=18)	Control (N=18)
Site, n(%)		
Oxford	15 (83.3%)	14 (77.8%)
Norfolk and Norwich	3 (16.7%)	4 (22.2%)
Spinal Accessory nerve sacrificed, n(%)		
Yes	1 (5.6%)	0 (0%)
No	17 (94.4%)	18 (100.0%)
Sex, n(%)		
Female	4 (22.2%)	5 (27.8%)
Male	14 (77.8%)	13 (72.2%)
Ethnicity, n(%)		
White	18 (100.0%)	18 (100.0%)
Handedness, n(%)		
Left	1 (5.6%)	0 (0%)
Right	16 (88.9%)	18 (100.0%)
Ambidexterity	1 (5.6%)	0 (0%)
Comorbidities, n(%)^*^		
Comorbidities list 1	4 (22.2%)	4 (22.2%)
Comorbidities list 2	12 (66.7%)	9 (50.0%)
Comorbidities list 3	0 (0%)	0 (0%)
Comorbidities list 4	6 (33.3%)	6 (33.3%)
Other	2 (11.1%)	4 (22.2%)
ASA Grade, n(%)		
1	7 (38.9%)	11 (61.1%)
2	6 (33.3%)	4 (22.2%)
3	4 (22.2%)	2 (11.1%)
4	1 (5.6%)	1 (5.6%)
In Work, n(%)		
In work (paid)	9 (50.0%)	12 (66.7%)
Not in work	9 (50.0%)	6 (33.3%)
Type of job, n(%)		
Sedentary occupation	5 (27.8%)	7 (38.9%)
Standing occupation	1 (5.6%)	1 (5.6%)
Physical work	3 (16.7%)	3 (16.7%)
Heavy manual work	0 (0%)	1 (5.6%)
Not Applicable	9 (50.0%)	6 (33.3%)
Smoker, n(%)		
Yes	3 (16.7%)	5 (27.8%)
No	15 (83.3%)	13 (72.2%)
**Years smoking, (n,Mean(SD))**	3, 46.7 (15.3)	5, 29.0 (14.7)
Alcohol drinker, n(%)		
Yes	13 (72.2%)	11 (61.1%)
No	5 (27.8%)	7 (38.9%)
**Alcohol units, (n,Mean(SD))**	11, 13.1 (8.8)	11, 21.7 (34.5)
**Age, (Mean(SD))**	60.1 (12.5)	62.2 (7.5)
**Neck and shoulder pain intensity, (n,Mean(SD))**	18, 1.9 (1.8)	18, 2.1 (1.8)
**Body Mass Index, (Mean(SD))**	29.2 (5.4)	28.4 (3.9)
**Pain relief medications, n(%)^†^**	11 (61.1%)	12 (66.7%)
Simple analgesic only	5 (27.8%)	5 (27.8%)
Complex analgesic only	4 (22.2%)	2 (11.1%)
Both analgesic	2 (11.1%)	5 (27.8%)
None	7 (38.9%)	6 (33.3%)
**Prior Chemotherapy or radiotherapy, n(%)**	3 (16.7%)	2 (11.1%)
**No prior treatment, n(%)**	15 (83.3%)	16 (88.9%)
Tumor pre-surgery location, n(%)		
Tonsil	5 (27.8%)	4 (22.2%)
Tongue base	5 (27.8%)	2 (11.1%)
Larynx	0 (0%)	0 (0%)
Hypopharynx	1 (5.6%)	1 (5.6%)
Skin	0 (0%)	1 (5.6%)
Salivary Glands (Submandibular)	2 (11.1%)	0 (0%)
Salivary Glands (Parotid)	2 (11.1%)	0 (0%)
Unknown primary	0 (0%)	3 (16.7%)
Oral Cavity	2 (11.1%)	5 (27.8%)
Clinical pre-surgery T-stage, n(%)		
T0	1 (5.6%)	1 (5.6%)
T1	5 (27.8%)	6 (33.3%)
T2	7 (38.9%)	4 (22.2%)
T3	1 (5.6%)	2 (11.1%)
T4	1 (5.6%)	3 (16.7%)
Missing	3 (16.7%)	2 (11.1%)
Clinical pre-surgery N stage, n(%)		
N0	4 (22.2%)	8 (44.4%)
N1	7 (38.9%)	5 (27.8%)
** N2**	2 (11.1%)	1 (5.6%)
N2a	0 (0%)	1 (5.6%)
N2b	1 (5.6%)	3 (16.7%)
N2c	1 (5.6%)	0 (0%)
Missing	3 (16.7%)	0 (0%)

Twenty-seven (75%) males and nine (25%) female participants were recruited with a mean age of 61 years (SD: 10.2). Twenty-one (58%) participants were in employment at the time of recruitment. Sixteen participants (44%) had tumours involving the oropharynx, tonsil and tongue base. Twenty-two (61%) participants had T1/2 tumours. Eight (22%) had undergone neck treatment in the preceding six months (surgery or radiotherapy). Five participants had prior chemotherapy or radiotherapy, (three intervention group; two control group).

The characteristics of surgical intervention are summarised in **Table 3**. The time to randomisation following ND was similar in both groups: experimental group mean 1.6 days (SD: 0.6) and the control group mean 1.9 days (SD: 1.5).

**Table 3:** Surgical characteristics of the cohort, illustrating neck dissections performed, levels dissected and damage/resection of key structures.

	Intervention		Control	
	Left (N=18), n(%)	Right (N=18), n(%)	Left (N=18), n(%)	Right (N=18), n(%)
Neck Dissection performed				
Yes	7 (38.9%)	12 (66.7%)	9 (50.0%)	13 (72.2%)
No	11 (61.1%)	6 (33.3%)	9 (50.0%)	5 (27.8%)
Level excised^*^				
1	4 (57.1%)	8 (66.7%)	6 (66.7%)	7 (53.8%)
2a	7 (100.0%)	12 (100.0%)	9 (100.0%)	11 (84.6%)
2b	7 (100.0%)	10 (83.3%)	7 (77.8%)	11 (84.6%)
3	7 (100.0%)	11 (91.7%)	9 (100.0%)	12 (92.3%)
4	7 (100.0%)	11 (91.7%)	5 (55.6%)	12 (92.3%)
5a	0 (0%)	2 (16.7%)	1 (11.1%)	0 (0%)
5b	0 (0%)	1 (8.3%)	1 (11.1%)	0 (0%)
6	0 (0%)	0 (0%)	0 (0%)	0 (0%)
Damage or resection of structures^*^				
SCM(sternocleidomastoid muscle)	0 (0%)	1 (8.3%)	0 (0%)	0 (0%)
IJV (Internal Jugular Vein)	0 (0%)	0 (0%)	0 (0%)	0 (0%)
Lingual Nerve	0 (0%)	0 (0%)	1 (11.1%)	0 (0%)
Marginal Mandibular nerve (VII)	0 (0%)	0 (0%)	0 (0%)	0 (0%)
Vagus nerve (X)	0 (0%)	0 (0%)	0 (0%)	0 (0%)
Accessory Nerve (XI)	0 (0%)	1 (8.3%)	0 (0%)	0 (0%)
Hypoglossal Nerve (XII)	0 (0%)	0 (0%)	0 (0%)	0 (0%)
Phrenic Nerve	0 (0%)	0 (0%)	0 (0%)	0 (0%)

*The proportions are calculated based on the number of participants with neck dissection performed. *Comorbidity list 1: Anxiety, Depression, Mental Illness.Comorbidity list2: Angina or heart condition, Asthma, Chronic lung disease, Peripheral vascular disease, Stroke, High blood pressure.Comorbidity list 3: Dementia, Parkinson’s disease, Multiple sclerosis.Comorbidity list 4: Arthritis, osteoporosis, diabetes, digestive problems.Comorbidity list 5: Cancer (other than HNC).†Simple pain relief medication: Aspirin, Ibuprofen, Paracetamol.Complex pain relief medication: Co-codamol, Codeine, Dihydrocodeine, Diclofenac, Gabapentin, Morphine, Naproxen, Pxycodone, Tramadol or other medications.

In both the experimental intervention and control groups, pathological lymph nodes were predominantly in level 2a/b. The **Supplementary Table 1** illustrates the levels involved in both groups. Patients in both groups were in hospital for a similar number of days; experimental group median 4.0 days and the control group median 5.0 days.

### Feasibility outcomes

#### Recruitment and retention

The trial identified 98 potential participants. However, due to COVID-19 pandemic, 23 (23.4%) identified potential participants did not complete the screening process. Out of the 75 screened participants, 56 (75%) were eligible, of which 39 (70%) consented to the trial. Due to COVID-19 or participants having died between consent and randomisation, three consented participants were excluded. Therefore 36 participants were successfully recruited. The trial was able to actively recruit for 18 months across two sites. The recruitment rate was one recruitment per site, per actively recruiting month, accounting for study pauses due to the COVID-19 pandemic.

The most common reason for potential participants being identified but not screened were: no further approach due to COVID-19 (30%), not followed-up due to insufficient staffing to approach (22%) or consent (17%). For those screened but ineligible, reasons for ineligibility were due to ND not planned as part of participant care (63%), follow-up by the participant being difficult due to not having access to the internet (21%). For those eligible but declined, the most common reason was participants not interested in taking part of research (41%) and distance of travel for follow-up (24%). The full list of reasons is included in **Supplementary Table 2**.

Of the 36 randomised participants, 26 completed the compulsory six months follow-up case report forms (CRFs). The retention rate to six months was 72% (95% CI: 58% to 87%).

#### Study intervention fidelity

**Figure 2:**
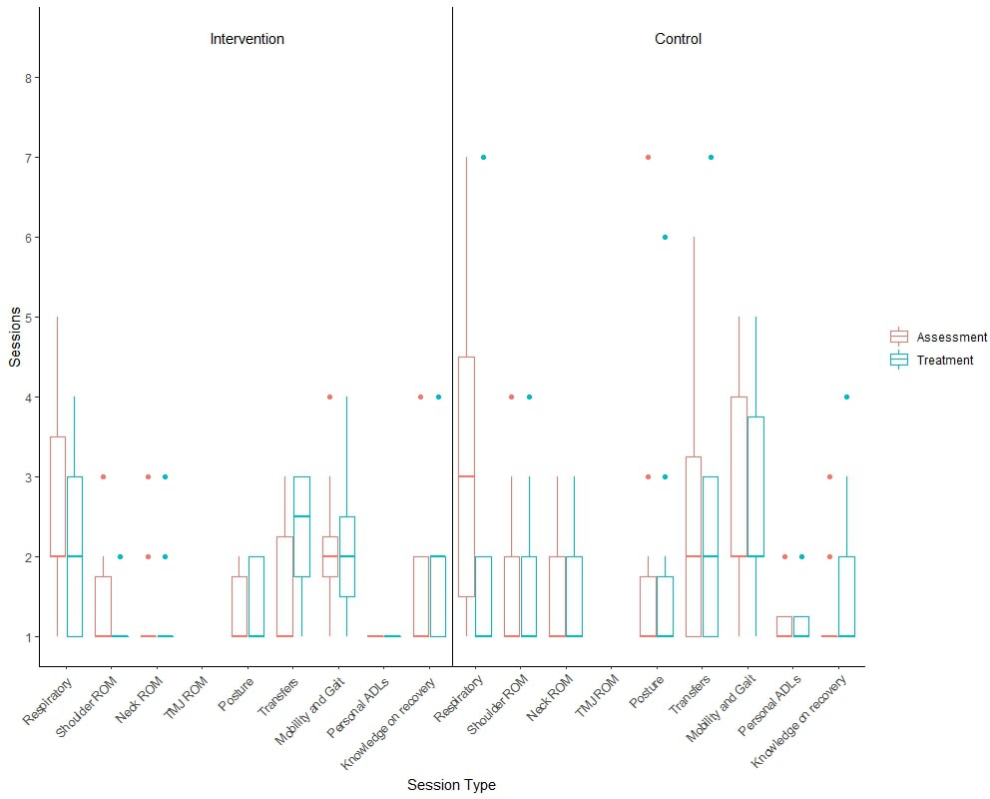
Boxplot illustrating the number of GRRAND sessions attended by participants for each of the in-patient programme.

**Table 4** shows the completion rates at six- and 12-month follow-up for key secondary outcomes. Participants in both treatment arms attended in-patient assessment and treatment programme before they were discharged from hospital. Participants in the intervention group attended a median of two sessions (IQR: 1.0, 3.8), while those in the control group attended a median of 2.5 sessions (IQR: 1.0, 4.8). **Figure 2** illustrates the range of rehabilitation interventions prescribed during these sessions.

**Table 4:** Data completeness at six and 12-months post-randomisation.

	6 Months			12 Months		
Completeness	Intervention(N=18), n(%)	Control (N=18), n(%)	Overall (N=36), n(%)	Intervention(N=9), n(%)	Control (N=9), n(%)	Overall (N=18), n(%)
**Death**	0 (0%)	0 (0%)	0 (0%)	0 (0%)	1 (11.1%)	1 (5.6%)
Withdrawal^*^	1 (5.6%)	1 (5.6%)	2 (5.6%)	0 (0%)	1 (11.1%)	1 (5.6%)
**SPADI**	14 (77.8%)	12 (66.7%)	26 (72.2%)	6 (66.7%)	5 (55.6%)	11 (61.1%)
**EQ5D**	14 (77.8%)	13 (72.2%)	27 (75.0%)	6 (66.7%)	5 (55.6%)	11 (61.1%)
**EPRTC QLQ C30**	14 (77.8%)	12 (66.7%)	26 (72.2%)	6 (66.7%)	5 (55.6%)	11 (61.1%)
**EORTC QLQ H&N43**	14 (77.8%)	13 (72.2%)	27 (75.0%)	6 (66.7%)	5 (55.6%)	11 (61.1%)
**Follow up questionnaire**	14 (77.8%)	13 (72.2%)	27 (75.0%)	6 (66.7%)	5 (55.6%)	11 (61.1%)

*One participant withdrew at 6 months follow up time point (if they were to stay on the trial) would not reach 12 months follow up time point when the trial ends, therefore we did not include them in the 12 months withdrawal section.

Participants randomised to the intervention arm received their first post-discharge physiotherapy session a mean of 9.6 days (SD: 4.9) from in-patient discharge. The number of sessions received by participants is presented in **Supplementary Table 3**. Due to the COVID-19 pandemic, participants could receive these intervention sessions either in hospital or virtually. The location and range of interventions is presented in **Supplementary Table 4**.

There were two withdrawals, one from the control arm and one from the experimental intervention arm. Both participants who withdrew did not provide reasons of their withdrawal. The participant in the experimental intervention arm withdrew before receiving any intervention. Accordingly, overall retention in the trial to six months was 92%.

### Data response rate

Due to COVID-19, many participants were not able to attend clinic and therefore did not have their physical performance measured at baseline and during follow-up visits. In the experimental intervention group, 14 had baseline measurements recorded, but at six and 12 months this had reduced to four and one participant respectively. In the control arm, 13 had baseline measurements. This reduced to seven and three participants at six and 12 months. The return rate is shown in **Table 4**. Of note, 16 participants reached the 12-month time-point. In summary, 72% to 75% of six-month PROMs data, dependent on questionnaire, were completed and returned.

### Clinical and patient-reported outcomes

The SPADI score ranges from 0 to 100, where 0 indicating the best and 100 indicating the worst shoulder function (22, 23). The summary statistics of SPADI scores at different time-points for the two treatment groups is displayed in **Supplementary Table 5**. There was a trend for the observed treatment differences in SPADI at six months to be in-favour of the intervention arm. The total SPADI score was a mean of -9.37 (95% CI: -20.66 to 1.93), whilst sub-scores were a mean of -0.09 (95% CI:-13.4 to 13.2) for pain and -13.56 (95%CI: -25.52, -1.60) for disability.

The results of the EORTC QLQ C30 and H&N43 (24, 25) are presented in **Supplementary Table 6**. In summary, those allocated to the experimental intervention demonstrated higher HRQoL scores across the domains compared to control group participants. This did not reach a statistically significant threshold as expected for this underpowered analysis.

### Progression criteria

**Table 5** presents the findings of the progression criteria analysis. As this indicates, the study design reached thresholds for feasibility for five of the six criteria. The study reached ‘green’ thresholds for consent where 70% of eligibility participants consented, intervention fidelity, where 78% of participants discharged completed the intervention sessions, contamination there no participants in the control group received the GRRAND intervention and retention where only 8% of participants were lost to follow-up. One criterion was categorised as ‘amber’ where 28% of participants demonstrated missing data in the questionnaire. Recruitment was the single criteria which was not met, with a ‘red’ outcome where only 36 from the originally 50 participants were recorded within 18 months. However, as stated earlier, this study was conducted throughout the COVID-19 pandemic where site activity was interrupted. Accordingly interpreting this criterion is challenging. The TOC on 20^th^ June 2022 were presented with the findings. They recommended, with modification, the trial was feasible.

**Table 5:** A summary of the results of the progression criteria based on the traffic-light stop-go criteria approach.

	Green (Go)	Amber (Amend)	Red (Stop)	Status	Implications of Findings
**Recruitment**	60 participants recruited within 12 months	40-59 participants recruited within 12 months	<40 participants recruited within 12 months	**Red** (due to COVID disruption) 36 participants were recruited within just 18 months of active recruitment.	On months when sites were staffed there were adequate potential participants numbers coming through clinics to recruit to target. Unfortunately, COVID continued to impact staff availability throughout.
**Consent**	≥40% of potentially eligible participants	20-39% of potentially eligible participants	<20% of potentially eligible participants	**Green** **70%** of eligible consented	
**GRRAND-F intervention fidelity**	>70% participants received protocol-compliant GRRAND-F intervention	50% to 70% received intervention as randomised	<50% received intervention as randomised	**Green** **78%** of participants discharged completed intervention sessions.	
**Contamination**	<5% participants in control group received GRRAND-F intervention	5-10% participants in control group received GRRAND-F intervention	>10% participants in control group received GRRAND-F intervention	**Green** **Nil** participants in control group received GRRAND-F intervention	
**Data Completion**	<15% missing data at 6-month follow-up	15-30% missing data	>30% missing data	**Amber** **28%** 6m FU questionnaires missing data	Based upon the PROMs data only, as clinical measures became optional after protocol amend no. 6.
**Retention**	<20% attrition at 6 month follow-up	20-50% attrition at 6 month follow-up	>50% attrition at 6 month follow-up	**Green** **8% attrition** (2 withdrawn and 1 lost to follow up)	

## Discussion

The findings of this study indicate that, whilst modifications may improve its efficiency, this proposed trial design for a pragmatic, multicentre RCT investigating the effectiveness and cost-effectiveness of a rehabilitation intervention on pain, function and HRQoL following ND for HNC is feasible. The findings also provide a signal that this intervention is potentially efficacious, certainly over a short-term, and at a level which is clinically significant. The impact of the COVID-19 pandemic on NHS site activities reduced the ability to test the design across the intended number of sites. Nonetheless, where tested, there were consistent data to indicate that both the approaches proposed to screen and consent participants in addition to collecting outcome data are suitable. Furthermore physiotherapists delivering the interventions demonstrated fidelity to the GRRAND intervention. As highlighted by the *a priori* progression criteria, there is strong evidence that this trial design would be feasible for the intervention to be tested in a full trial.

The trial design indicated modification to two key aspects. Firstly, whilst the return of CRFs was within intended expectations, the return of intervention exercises diaries was low (50%). The use of exercise diaries in this trial design was to ascertain adherence levels between rehabilitation sessions. Whilst the embedded qualitative study indicated adherence was good from study participants (16), the numerical data which we anticipated could accompany this, was lacking. Exercise diaries and assessment of rehabilitation adherence has been acknowledged as a major challenges in other trials (26). Given this, and the pragmatic nature of the trial design, we propose a ‘light-touch’ assessment to exercise diary data where participants in the full trial may indicate adherence and compliance to the intervention through attendance in physiotherapy, which was well-collected in the health-resource use questionnaire. Secondly, as a result of the COVID-19 pandemic, a number of participants attended follow-up appointments remotely. It is anticipated that this may continue longer-term with online consultations in the future (27). The change from face-to-face to remote consultations resulted in an inability to collect physical function measures, notably joint range of motion and handheld dynamometry assessments. As the SPADI includes elements of physical performance and capability within its subsections, it is proposed that assessing physical function through such a PROM rather than physical function may not only improve the flexibility of collecting this domain, as not reliant on face-to-face consultations, but may be more time and cost-effective by collecting *via* post or online rather than requiring transport and associated costs. Furthermore, as a number of patients receive their surgery within tertiary centres in the NHS for HNC ND, the travel to these specialist centres can be considerable. Accordingly, collecting such data remotely may reduce the burden on these patients, particularly during a potentially stressful healthcare episode following ND.

This study presented with a number of major successes. Firstly, there was clear support from participants for the design and conduct of this study. This is evidenced with our high conversion rate between eligible participant approach to consent (70%). Furthermore, the participant attitudes towards the GRRAND intervention, as reported in (16), further augment this notion. Secondly, given the challenges in managing site opening and research conduct during the COVID-19 pandemic, the ability to undertake this across two NHS hospitals was a major success. However, as a weakness, due to this reason we were unable to open the planned further two sites and recruited 24 fewer participants than originally planned (19). These sites would have offered further learning on the study design to supplementary the findings reported in this study. Finally, as a result of the pandemic, the planned face-to-face data collection processes were not implemented throughout. However, this opportunity meant we were agile and able to make protocol amendments to ensure not only the intervention delivery could be delivered virtually, but also data collection could be modified for this eventually. The benefit of this meant we now have the knowledge on how to adopt both face-to-face or virtual approaches for intervention delivery and data collection mechanisms to the benefit of designing a full trial. Finally, the result indicate heterogeneity in pre-operative status and adjunctive treatments across the cohort. Whilst there is a risk that this differed between the groups for this small sample, it is anticipated that equivalence would be achieved with a larger cohort. Consideration should be made on whether these are important prognostic factors to warrant inclusion as part of the minimisation randomisation procedure for a full-trial.

During the design of this feasibility study, we did not stipulate a proposed primary outcome measure for a full trial. Armed with the evidence from this feasibility study, based on high data returns and the ability to collect multiple domains from the same PROM, the SPADI would appear to be an appropriate instrument. This has been endorsed by our patient and public involvement in research members. Based on this, the estimated sample size for a future trial with SPADI as primary outcome is 416, assuming participants are randomised 1:1 using a clinically meaningful difference of eight points in the SPADI from the GRASP trial (26), SD of 21.7 scores, 90% power, 5% significance level and 20% attrition rate. Alternatively the estimated sample sizes for a future trial with EORTC C-30 global health status as primary outcome are 588 or 376, based on the estimated minimal difference from a group study which investigated six EORTC trials (28). This information will form the basis of our plans for the full-trial to test the GRRAND intervention.

## Conclusions

The findings from this feasibility study indicate the proposed trial design for a pragmatic, multicentre RCT of testing a rehabilitation intervention following ND for HNC was assessed as feasible with modifications. A full-trial, based on these findings, can now be designed to better understand whether this proposed rehabilitation interventions is clinically and cost-effective for this population. This remains important as given the increase in HNC prevalence in younger patients, these individuals increasingly require an evidence-based rehabilitation programme to reduce morbidly and improve functional performance after this surgical procedure.

## Data availability statement

The original contributions presented in the study are included in the article/**Supplementary Material**. Further inquiries can be directed to the corresponding author.

## Ethics statement

The studies involving human participants were reviewed and approved by South Central - Oxford B Research Ethics Committee. IRAS project ID: 268845. The patients/participants provided their written informed consent to participate in this study.

## Author contributions

The author list comprises the study group responsible for administration and execution of the trial. TS, SD, SL, and SW were responsible for conception, design and execution of the study; AG, AM, and VG, were responsible for administrative management of the study; BF was responsible for qualitative research; SD, TL and MCJ were responsible for statistics. All authors contributed to the article and approved the submitted version.
