# Supplementary material for: Getting Recovery Right After Neck Dissection (GRRAND-F): Mixed-methods feasibility study to design a pragmatic randomised controlled trial
Source: Front Oncol. 2023 Mar 16;13:1110500. doi: 10.3389/fonc.2023.1110500 (PMC10061058; doi:10.3389/fonc.2023.1110500)
Supplement: Supplementary file 1 [file DataSheet_1.docx]

**SUPLEMENTARY FILE 1**: Level of nodal metastasis

|  | **Intervention** | | **Control** | |
| --- | --- | --- | --- | --- |
|  | **Left(N=18),   n(%)** | **Right(N=18),  n(%)** | **Left(N=18),  n(%)** | **Right(N=18),  n(%)** |
| **Node level with metastasis** |  |  |  |  |
| 1 | 0 (0%) | 2 (11.1%) | 0 (0%) | 0 (0%) |
| 2a | 2 (11.1%) | 5 (27.8%) | 3 (16.7%) | 4 (22.2%) |
| 2b | 1 (5.6%) | 2 (11.1%) | 0 (0%) | 1 (5.6%) |
| 3 | 1 (5.6%) | 4 (22.2%) | 1 (5.6%) | 3 (16.7%) |
| 4 | 0 (0%) | 1 (5.6%) | 0 (0%) | 1 (5.6%) |
| 5a, 5b and 6 | 0 (0%) | 0 (0%) | 0 (0%) | 0 (0%) |

**SUPLEMENTARY FILE 2**: Primary reasons of decline in each stage of screening and randomisation

| **Identified but not screened** | **N=23** |
| --- | --- |
| No further approach due to COVID-19 | 7 (30.4%) |
| Insufficient staff to approach patients^*^ | 5 (21.7%) |
| Insufficient staff to consent patients^*^ | 4 (17.4%) |
| Insufficient physio staff to treat | 3 (13.0%) |
| Funding extension still pending at the time^*^ | 2 (8.7%) |
| Patient attended clinic not covered by research staff | 1 (4.3%) |
| Patient very tearful in consultation | 1 (4.3%) |
| **Largely due to COVID-19* | |
| **Screened but not eligible** | **N=19** |
| Neck dissection not planned part of participant care | 12 (63.2%) |
| Patient does not have access to internet | 4 (21.1%) |
| Patient has significant difficulties reading and/or comprehensive English | 2 (10.5%) |
| History of pre-existing, long term neurological disease affecting the shoulder | 1 (5.3%) |
|  |  |
| **Eligible but declined to participate** | **N=17** |
| Not interested in taking part of research | 7 (41.2%) |
| Distance of travel | 4 (2.5%) |
| Insufficient time to approach in clinic (patient wished to leave after appointment) | 1 (5.9%) |
| Caring responsibility | 1 (5.9%) |
| Too busy | 1 (5.9%) |
| Unable to attend sessions | 1 (5.9%) |
| Leaving the area | 1 (5.9%) |
| Prefer not to say | 1 (5.9%) |
|  |  |
| **Consented but not randomised** | **N=3** |
| No further approach due to COVID-19 | 2 |
| Death | 1 |

**SUPLEMENTARY FILE 3**: Summary of intervention session received by participants randomised to intervention session

|  | **Intervention (N=18)** |
| --- | --- |
| **Number of intervention sessions patients attended (n, Mean(SD))** | 16, 4.1 (1.6) |
| 0 | 2 (11.1%) |
| 1 | 2 (11.1%)^*^ |
| 2 | 0 (0%) |
| 3 | 3 (16.7%) |
| 4 | 5 (27.8%) |
| 5 | 2 (11.1%) |
| 6 | 4 (22.2%) |
| **Time (in Days) from discharge to receiving the first intervention session (n, Mean(SD)** | 9, 9.6 (4.9) |
| *^*^One participant on the intervention arm withdrew before receiving their first intervention*  *One participant did not receive any interventions due to COVID-19* | |

**SUPLEMENTARY FILE 4**: Intervention sessions received by participants randomised to intervention arm

| **Only for intervention arm (N=18) ^*^** | **Session 1** | **Session 2** | **Session 3** | **Session 4** | **Session 5** | **Session 6** |
| --- | --- | --- | --- | --- | --- | --- |
| **Attended the intervention session^$^** | 16 (88.9%) | 14 (77.8%) | 14 (77.8%) | 10 (55.6%) | 7 (38.9%) | 4 (22.2%) |
| **Location** |  |  |  |  |  |  |
| Hospital Clinic | 11 (68.8%) | 9 (64.3%) | 11 (78.6%) | 7 (70.0%) | 5 (71.4%) | 3 (75.0%) |
| IP ward | 1 (6.2%) | 0 (0%) | 0 (0%) | 0 (0%) | 0 (0%) | 0 (0%) |
| Video consultation | 4 (25.0%) | 5 (35.7%) | 3 (21.4%) | 3 (30.0%) | 2 (28.6%) | 1 (25.0%) |
| Community Clinic | 0 (0%) | 0 (0%) | 0 (0%) | 0 (0%) | 0 (0%) | 0 (0%) |
| Home-based | 0 (0%) | 0 (0%) | 0 (0%) | 0 (0%) | 0 (0%) | 0 (0%) |
| Not Applicable |  |  |  |  |  |  |
| **Intervention type** |  |  |  |  |  |  |
| Subjective Assessment | 16 (100.0%) | 14 (100.0%) | 14 (100.0%) | 9 (90.0%) | 7 (100.0%) | 4 (100.0%) |
| Objective Assessment | 16 (100.0%) | 14 (100.0%) | 13 (92.9%) | 9 (90.0%) | 6 (85.7%) | 3 (75.0%) |
| TMJ ROM | 2 (12.5%) | 2 (14.3%) | 1 (7.1%) | 1 (10.0%) | 1 (14.3%) | 0 (0%) |
| Cervical ROM | 13 (81.2%) | 13 (92.9%) | 13 (92.9%) | 8 (80.0%) | 5 (71.4%) | 3 (75.0%) |
| ScapularROM | 14 (87.5%) | 9 (64.3%) | 12 (85.7%) | 8 (80.0%) | 4 (57.1%) | 2 (50.0%) |
| GHJ ROM | 13 (81.2%) | 11 (78.6%) | 11 (78.6%) | 8 (80.0%) | 6 (85.7%) | 2 (50.0%) |
| TMJ PRE | 0 (0%) | 0 (0%) | 0 (0%) | 0 (0%) | 0 (0%) | 0 (0%) |
| Cervical PRE | 1 (6.2%) | 1 (7.1%) | 0 (0%) | 1 (10.0%) | 1 (14.3%) | 1 (25.0%) |
| Shoulder PRE | 12 (75.0%) | 9 (64.3%) | 10 (71.4%) | 6 (60.0%) | 6 (85.7%) | 3 (75.0%) |
| Limb Positioning | 8 (50.0%) | 3 (21.4%) | 2 (14.3%) | 3 (30.0%) | 1 (14.3%) | 1 (25.0%) |
| Oral Health | 4 (25.0%) | 1 (7.1%) | 1 (7.1%) | 1 (10.0%) | 0 (0%) | 0 (0%) |
| Pain Management | 6 (37.5%) | 4 (28.6%) | 3 (21.4%) | 5 (50.0%) | 3 (42.9%) | 1 (25.0%) |
| Scar Management | 14 (87.5%) | 12 (85.7%) | 12 (85.7%) | 9 (90.0%) | 5 (71.4%) | 1 (25.0%) |
| Exercise Adherence Discussion | 9 (56.2%) | 10 (71.4%) | 10 (71.4%) | 7 (70.0%) | 6 (85.7%) | 3 (75.0%) |
| Pacing & Behaviours Modification Discussion | 11 (68.8%) | 8 (57.1%) | 8 (57.1%) | 7 (70.0%) | 4 (57.1%) | 0 (0%) |
| Independence & Return to ADL/Work/Role Discussion | 9 (56.2%) | 5 (35.7%) | 7 (50.0%) | 6 (60.0%) | 5 (71.4%) | 3 (75.0%) |
| Other interventions | 12 (75.0%) | 11 (78.6%) | 12 (85.7%) | 6 (60.0%) | 5 (71.4%) | 3 (75.0%) |
| *^*^One participant on the intervention arm withdrew before receiving their first intervention*  *^$*^The proportions are calculated based on the number of participants attended that session* | | | | | | |

**SUPLEMENTARY FILE 5**: SPADI scores summary

|  | **Intervention (N=18)** | **Control (N=18)** | **Overall (N=36)** | **Overall Treatment difference at 6 months (95% CI)^*^** |
| --- | --- | --- | --- | --- |
| **SPADI Pain Score (N,Mean(SD))** |  |  |  | -0.092,(-13.39 , 13.206) |
| Baseline | 18, 6.6 (13.2) | 18, 6.0 ( 9.0) | 36, 6.3 (11.2) |  |
| 6 months | 14, 20.7 (22.6) | 12, 26.0 (26.0) | 26, 23.2 (23.9) |  |
| 12 months^†^ | 6, 13.0 (24.3) | 5, 23.2 (26.8) | 11, 17.6 (24.7) | -1.954,(-21.743 , 17.835) |
| **SPADI Disability Score (N,Mean(SD))** |  |  |  | -13.559,(-25.523 , -1.595) |
| Baseline | 18, 5.1 (14.3) | 18, 0.7 ( 1.9) | 36, 2.9 (10.3) |  |
| 6 months | 14, 12.1 (22.0) | 12, 18.3 (21.3) | 26, 15 (21.5) |  |
| 12 months^†^ | 6, 4.0 ( 7.9) | 5, 21.2 (24.9) | 11, 11.8 (19) | -16.272,(-34.772 , 2.228) |
| **Total SPADI Score (N,Mean(SD))** |  |  |  | -9.365,(-20.659 , 1.928) |
| Baseline | 18, 5.6 (12.6) | 18, 2.7 ( 3.8) | 36, 4.2 (9.3) |  |
| 6 months | 14, 15.4 (21.8) | 12, 21.3 (22.0) | 26, 18.1 (21.7) |  |
| 12 months^†^ | 6, 7.4 (14.2) | 5, 22.0 (25.4) | 11, 14.1 (20.4) | -10.818,(-28.404 , 6.768) |
| *^*^ The overall treatment difference is estimated based on SPADI at 6 months, it is calculated by linear model with SPADI at 6 months (only) as outcome and treatment allocation as independent variable, the model is adjusted only on baseline SPADI score.* | | | | |
| *^†^Only 18 participants reached the 12 months follow up period, of which 9 were in the intervention arm and 9 were in the control arm.*  *The treatment difference at 12 months it is calculated by linear model with SPADI as outcome and treatment allocation and follow-up timepoint interaction as independent variable, the model is adjusted only on baseline SPADI score.* | | | | |

**SUPLEMENTARY FILE 6**: Summary of the EORTC QLQ C30 and H&N43 scores at baseline, six and 12 months (for those who reached 12 month follow-up) for control and intervention group.

|  | **Baseline** | | **6 Months** | | **12 Months** | |  |  |
| --- | --- | --- | --- | --- | --- | --- | --- | --- |
|  | **Intervention(N=18),   n(%)** | **Control(N=18),  n(%)** | **Intervention(N=18),  n(%)** | **Control(N=18),  n(%)** | **Intervention(N=9),   n(%)** | **Control(N=9),  n(%)** | **Overall Treatment difference at 6 months (95% CI)^*^** | **Treatment difference at 12 months(95% CI)** **^†^** |
| **QLQ-C30 (N,Mean(SD))** |  |  |  |  |  |  |  |  |
| Global health status | 17, 74.5 (28.3) | 18, 70.4 (20.1) | 14, 72.6 (28.9) | 13, 59.0 (20.8) | 6, 68.1 (19.3) | 5, 50.0 (37.3) | 18.751,(7.383 , 30.119) | -5.782,(-33.217 , 21.653) |
| **Functional scale** |  |  |  |  |  |  |  |  |
| Physical functioning | 17, 89.8 (22.6) | 18, 91.5 (10.2) | 14, 84.8 (18.0) | 12, 82.8 (20.4) | 6, 95.6 ( 6.9) | 5, 73.3 (29.4) | 5.318,(-5.668 , 16.303) | 16.676,(-1.529 , 34.881) |
| Role functioning | 18, 88.0 (29.6) | 18, 88.9 (19.0) | 14, 84.5 (31.0) | 12, 80.6 (25.5) | 6, 80.6 (32.3) | 5, 70.0 (41.5) | 6.474,(-12.52 , 25.469) | 0.759,(-32.868 , 34.386) |
| Emotional functioning | 18, 76.9 (29.6) | 18, 74.5 (22.8) | 14, 78.0 (29.0) | 13, 78.2 (32.4) | 6, 80.6 (22.8) | 5, 50.0 (43.7) | 0.487,(-12.279 , 13.253) | 4.2,(-20.858 , 29.257) |
| Cognitive functioning | 18, 84.3 (25.9) | 18, 88.9 (19.8) | 14, 76.2 (29.8) | 13, 83.3 (23.6) | 6, 86.1 (19.5) | 5, 66.7 (39.1) | 0.532,(-9.46 , 10.524) | 0.878,(-21.164 , 22.919) |
| Social functioning | 18, 89.8 (21.5) | 18, 79.6 (27.7) | 14, 76.2 (35.0) | 13, 69.2 (33.2) | 6, 77.8 (25.1) | 5, 56.7 (52.2) | 4.057,(-19.057 , 27.17) | 9.128,(-28.189 , 46.445) |
| **Symptom scale** |  |  |  |  |  |  |  |  |
| Fatigue | 18, 21.6 (30.4) | 18, 23.5 (32.1) | 14, 23.8 (26.5) | 12, 32.4 (27.0) | 6, 22.2 (12.2) | 5, 42.2 (31.8) | -10.958,(-28.776 , 6.86) | -12.751,(-42.127 , 16.625) |
| nausea and vomiting | 18, 3.7 (9.1) | 18, 2.8 (6.4) | 14, 4.8 (10.2) | 12, 4.2 (10.4) | 6, 2.8 (6.8) | 5, 10.0 (9.1) | -1.642,(-4.071 , 0.786) | -3.692,(-9.716 , 2.332) |
| Pain | 18, 19.4 (35.8) | 18, 25.9 (34.9) | 13, 14.1 (16.5) | 12, 31.9 (27.9) | 6, 25.0 (32.9) | 5, 36.7 (44.7) | -13.876,(-29.587 , 1.836) | 0.202,(-35.353 , 35.757) |
| Dyspnoea | 18, 13.0 (20.3) | 18, 11.1 (22.9) | 14, 9.5 (20.4) | 12, 16.7 (33.3) | 6, 5.6 (13.6) | 5, 20.0 (18.3) | -11.841,(-29.844 , 6.162) | -5.71,(-34.176 , 22.755) |
| Insomnia | 18, 33.3 (36.2) | 18, 27.8 (34.8) | 14, 31.0 (42.3) | 12, 38.9 (37.2) | 6, 27.8 (39.0) | 5, 33.3 (40.8) | -13.14,(-43.323 , 17.044) | -7.771,(-54.3 , 38.757) |
| Appetite loss | 18, 11.1 (25.6) | 18, 14.8 (30.7) | 14, 14.3 (25.2) | 12, 16.7 (30.2) | 6, 22.2 (27.2) | 5, 13.3 (18.3) | -2.497,(-23.045 , 18.052) | 15.6,(-16.228 , 47.429) |
| Constipation | 18, 18.5 (36.6) | 18, 13.0 (23.3) | 14, 4.8 (12.1) | 12, 19.4 (26.4) | 6, 27.8 (39.0) | 5, 13.3 (29.8) | -15.592,(-30.742 , -0.443) | 18.156,(-11.898 , 48.21) |
| Diarrhea | 18, 3.7 (15.7) | 18, 3.7 (10.8) | 14, 2.4 ( 8.9) | 12, 5.6 (13.0) | 6, 0.0 (0.0) | 5, 0.0 (0.0) | -3.68,(-12.47 , 5.111) | 0,(-11.2 , 11.2) |
| Financial Difficulties | 18, 3.7 (15.7) | 18, 5.6 (17.1) | 14, 7.1 (19.3) | 13, 7.7 (27.7) | 6, 0.0 ( 0.0) | 5, 40.0 (54.8) | -1.659,(-19.12 , 15.802) | -34.923,(-66.27 , -3.577) |
| **QLQ H&N43** |  |  |  |  |  |  |  |  |
| Anxiety | 18, 30.6 (27.0) | 18, 52.8 (33.0) | 14, 39.3 (33.7) | 13, 47.4 (32.5) | 6, 22.2 (17.2) | 5, 60.0 (48.0) | -0.067,(-19.876 , 19.742) | -16.385,(-53.504 , 20.734) |
| Body image | 18, 8.6 (15.0) | 18, 12.3 (19.0) | 13, 21.4 (25.0) | 13, 35.0 (37.4) | 6, 14.8 (16.7) | 5, 44.4 (51.5) | -9.818,(-33.187 , 13.552) | -10.839,(-50.263 , 28.586) |
| Coughing | 18, 11.1 (19.8) | 18, 9.3 (15.4) | 14, 7.1 (19.3) | 13, 17.9 (25.9) | 6, 27.8 (32.8) | 5, 40.0 (36.5) | -9.468,(-26.319 , 7.384) | -7.021,(-37.162 , 23.121) |
| Dry mouth and sticky saliva | 18, 17.6 (24.6) | 18, 17.6 (20.2) | 14, 25.0 (25.9) | 13, 41.0 (30.1) | 6, 36.1 (24.5) | 5, 73.3 (43.5) | -15.777,(-37.075 , 5.522) | -33.334,(-69.174 , 2.505) |
| Neurological problems | 18, 11.1 (22.9) | 18, 13.0 (20.3) | 14, 31.0 (42.3) | 13, 23.1 (39.4) | 6, 11.1 (17.2) | 5, 40.0 (43.5) | 8.464,(-20.882 , 37.809) | -19.189,(-62.567 , 24.189) |
| Opening mouth | 18, 7.4 (24.4) | 18, 5.6 (17.1) | 14, 23.8 (33.1) | 13, 23.1 (39.4) | 6, 11.1 (17.2) | 5, 53.3 (50.6) | -0.668,(-24.74 , 23.405) | -24.787,(-62.119 , 12.545) |
| Pain in the head and neck | 18, 25.0 (32.2) | 18, 32.9 (35.3) | 14, 25.0 (24.0) | 13, 28.8 (23.5) | 6, 15.3 (15.3) | 5, 31.7 (19.0) | -3.296,(-20.104 , 13.512) | -5.502,(-31.258 , 20.253) |
| Social contact | 18, 11.1 (32.3) | 17, 9.8 (25.7) | 14, 7.1 (14.2) | 13, 20.5 (37.4) | 6, 5.6 (13.6) | 5, 26.7 (43.5) | -13.408,(-34.851 , 8.034) | -20.891,(-55.425 , 13.644) |
| Problems with senses | 18, 8.3 (24.4) | 18, 8.3 (13.1) | 14, 16.7 (20.7) | 13, 21.8 (20.8) | 6, 33.3 (40.8) | 5, 3.3 ( 7.5) | -4.007,(-20.099 , 12.085) | 21.978,(-4.904 , 48.86) |
| Shoulder problems | 18, 8.3 (16.4) | 18, 6.5 (11.6) | 13, 21.8 (23.0) | 13, 29.5 (35.5) | 6, 16.7 (33.3) | 4, 16.7 (33.3) | -6.496,(-28.068 , 15.076) | -1.65,(-36.911 , 33.611) |
| Skin problems | 18, 11.1 (20.9) | 18, 4.3 (10.9) | 14, 14.3 (25.9) | 13, 12.0 (24.2) | 6, 7.4 (11.5) | 5, 20.0 (27.7) | 3.412,(-16.628 , 23.452) | -12.709,(-41.627 , 16.209) |
| Swelling in the neck | 18, 25.9 (35.3) | 18, 22.2 (32.3) | 14, 31.0 (35.7) | 13, 20.5 (21.7) | 6, 16.7 (27.9) | 5, 26.7 (36.5) | 12.542,(-8.989 , 34.074) | -14.06,(-48.376 , 20.256) |
| Social eating | 17, 10.3 (25.4) | 16, 15.6 (22.7) | 14, 19.6 (17.2) | 13, 40.4 (30.4) | 6, 13.9 (10.1) | 5, 46.7 (35.6) | -22.636,(-41.688 , -3.584) | -22.737,(-52.046 , 6.572) |
| Speec problems | 18, 9.6 (20.6) | 17, 15.7 (24.6) | 14, 18.1 (20.0) | 13, 28.7 (28.9) | 6, 7.8 (12.9) | 5, 32.0 (31.8) | -7.064,(-24.804 , 10.676) | -19.941,(-45.537 , 5.654) |
| Problems with swallowing | 18, 7.4 (17.6) | 18, 16.2 (24.7) | 14, 11.3 (16.5) | 13, 19.2 (28.1) | 6, 13.9 (16.4) | 5, 33.3 (35.8) | -5.248,(-22.225 , 11.729) | -9.513,(-36.035 , 17.009) |
| Problems with sexuality | 17, 13.7 (26.5) | 18, 24.1 (37.6) | 14, 26.2 (36.8) | 12, 33.3 (35.5) | 6, 25.0 (23.0) | 5, 50.0 (50.0) | -2.648,(-27.523 , 22.226) | -8.275,(-51.352 , 34.803) |
| Problems with teeth | 18, 9.9 (18.6) | 18, 16.7 (25.6) | 14, 15.1 (22.9) | 13, 29.1 (30.6) | 6, 0.0 ( 0.0) | 5, 57.8 (34.6) | -10.675,(-26.695 , 5.345) | -40.372,(-69.011 , -11.733) |
| Weight loss | 18, 9.3 (27.5) | 18, 3.7 (10.8) | 14, 14.3 (25.2) | 13, 7.7 (20.0) | 6, 5.6 (13.6) | 5, 20.0 (44.7) | 3.395,(-10.489 , 17.278) | -14.444,(-41.329 , 12.44) |
| Problems with wound healing | 17, 9.8 (25.7) | 18, 13.0 (28.3) | 14, 9.5 (27.5) | 13, 5.1 (18.5) | 6, 0.0 ( 0.0) | 5, 20.0 (44.7) | 5.894,(-12.847 , 24.636) | -18.49,(-49.6 , 12.62) |

| *^*^ The overall treatment difference is estimated based on EORTC score at 6 months, it is calculated by linear model with EORTC score at 6 months (only) as outcome and treatment allocation as independent variable, the model is adjusted only on baseline EORTC score.* |
| --- |
| *^†^Only 18 participants reached the 12 months follow up period, of which 8 were in the intervention arm and 8 were in the control arm.*  *The treatment difference at 12 months it is calculated by linear model with EORTC score as outcome and treatment allocation and follow-up timepoint interaction as independent variable, the model is adjusted only on baseline EORTC score.* |

EORTC QLQ C30 and H&N43For the functional scale, a higher score indicates better level of functioning. For the global health status, a higher score indicates high quality of life. For the symptom scale, a higher score represents high level of problems (therefore worse symptom). The scales in H&N43 questionnaire follows the symptom scale score interpretation, the higher the score, the worse the problem. The observed treatment difference at 6 months for each scale is shown.
